# Drug-Induced Liver Injury Induced by Bioflavonoids

**DOI:** 10.7759/cureus.20453

**Published:** 2021-12-16

**Authors:** João P Pais, Rita Mota, Mariana Cruz, Ana R Cambão, Ana Nascimento

**Affiliations:** 1 Internal Medicine, Unidade Local de Saúde do Alto Minho (ULSAM) - Hospital Santa Lúzia, Viana do Castelo, PRT; 2 Pathology, Unidade Local de Saúde do Alto Minho (ULSAM) - Hospital Santa Lúzia, Viana do Castelo, PRT

**Keywords:** cholestatic liver injury, acute liver injury, idiosyncratic dili, bioflavonoids, drug-induced liver injury

## Abstract

Drug-induced liver injury (DILI) is a relatively rare disease with a vast spectre of clinical manifestations. Its diagnosis is based on the exclusion of other causes of liver disease. The identification of a causal agent is based on the temporal relation between the symptoms and their resolution after stopping the suspected drug. Many drugs have been described as causative agents of DILI; however, bioflavonoids have never been implied with an idiosyncratic DILI in the literature.

## Introduction

Drug-induced liver injury (DILI) is a relatively rare condition with an estimated incidence between 2.3 and 13.9 cases per 100000 persons in Europe [1]. However, it is a disease with a high impact in terms of morbidity and mortality, as it is one of the main causes of acute hepatic failure in the United States and Europe [2,3], and has an important socio-economic role as it is the main event responsible for the withdrawal of drugs from the market [2]. Typically, DILI is classified as intrinsic (or direct) or idiosyncratic [1,2,4]. Intrinsic DILI is dose-related, with an onset of hours to a few days after the use of the causative agent [2,4]. Because a high proportion of patients develop the same reaction to exposure to a certain drug, intrinsic DILI is also described as a predictable reaction [4]. Acetaminophen is the classic example of an intrinsic DILI and is responsible for about half the cases of acute liver injury in the US [1]. On the other hand, idiosyncratic DILI is an unpredictable reaction, is not dose-related (although a dose of 50-100 mg of the implied drug is usually required), occurs in a small proportion of exposed individuals, and has a variable latency period after exposure (from days to weeks) [1,3-5]. Many different drugs have been implied in DILI cases, however, there is no description of cases of DILI related to the use of bioflavonoids, typically used for chronic venous insufficiency treatment.

This work was presented as an ePoster in the 26th Portuguese Congress of Internal Medicine, which took place in Braga, between 27th and 30th August of 2020.

## Case presentation

We present the case of an 85-year-old female with a medical history of thromboembolic pulmonary hypertension (group 4) with secondary heart failure with preserved ejection fraction and hepatic congestion, usually in a functional class II of the New York Heart Association (NYHA), permanent atrial fibrillation, anticoagulated with warfarin, dyslipidaemia, and a history of cholecystectomy due to acute cholecystitis. Her usual medication, in addition to warfarin, was furosemide (40 mg, two times a day), spironolactone (50 mg, daily), and atorvastatin (20 mg, daily). She presented to the emergency department (ED) with aggravated breathlessness, with complaints for minimum efforts (functional class III of NYHA), peripheral oedema, and abdominal distension with about two weeks of evolution. Associated with these complaints, she also referred asthenia, anorexia, and non-quantified weight loss. On physical examination, she had jugular venous pressure at 45º, inspiratory crackles in the lower halves of both lungs, and bilateral peripheral oedema.

The blood tests revealed a cholestatic pattern of the hepatic enzymes, with elevation of total and direct bilirubin, alkaline phosphatase (ALP), and gamma-glutamyltransferase (GGT). The complete blood tests are summarized in Table 1. An abdominal ultrasound revealed the presence of grade 1 ascites and augmented hepatic echogenicity with prominent hepatic veins, suggestive of congestive hepatopathy. The symptoms were interpreted in the context of decompensated heart failure. Intravenous (IV) diuretic treatment with furosemide was started, with significant clinical improvement. The patient was discharged to the outpatient clinic after one day of observation. The blood tests were not revaluated prior to discharge.

**Table 1:** Blood test results on the first emergency department observation, revealing a cholestatic pattern of the hepatic enzymes.

	Results	Reference values
Haemoglobin	15.6 g/dL	11.8 - 15.8 g/dL
Leukocytes	8.05x10^9^/L	4-10x10^9^/L
Platelets	136x10^9^/L	150-400x10^9^/L
Urea	43 mg/dL	17-43 mg/dL
Creatinine	0.75 mg/dL	0.7-1.0 mg/dL
Sodium	138 mmol/L	136-145 mmol/L
Potassium	4.4 mmol/L	3.5-5.1 mmol/L
Total bilirubin	2.4 mg/dL	0.3-1.2 mg/dL
Direct bilirubin	1.25 mg/dL	<0.5 mg/dL
Alkaline Phosphatase	214 IU/L	30-120 IU/L
Gamma-glutamyltransferase	425 IU/L	<38 IU/L
Aspartate Transaminase	26 IU/L	8-35 IU/L
Alanine transaminase	21 IU/L	7-45 IU/L
Albumin	4.4 g/dL	3.5-5-2 g/dL
International Normalized Ratio (INR)	2.05	

Two months after the described episode, the patient was seen in the outpatient clinic with complaints of aggravating peripheral oedema, without any other complaints, which was interpreted as peripheral venous insufficiency. The patient was medicated with bioflavonoids, 500 mg twice a day. After one week of treatment, she noticed jaundice, which she associated with the start of the new medication, suspending it by her own initiative. One week after the stoppage of bioflavonoid treatment, the jaundice started to regress and disappeared after two weeks. 

Two months after the jaundice episode, peripheral oedema reappeared, and the patient decided by her initiative to restart treatment with bioflavonoids. About two weeks after the start of the drug, jaundice of the skin and sclera appeared, associated with generalized pruritus, choluria, and acholia. With these symptoms, bioflavonoids were once again stopped, but no clinical improvement was noted by the patient, which motivated a new observation in the ED. During this period, the patient negated consumption of herbal medicines, consumption of alcohol or other drugs, recent abroad trips or contact with animals, or changes of dosage of current medication.

On physical examination, jaundice of the skin and sclera was evident. There was no observable jugular venous pressure. Arterial pressure was 117/67 mmHg. The patient was apyretic, with an auricular temperature of 36.1ºC. Cardiac auscultation was arrhythmic, without other alterations, and pulmonary auscultation revealed very discrete crackles in both pulmonary bases. In the abdominal examination, no organomegalies or masses were palpable and there were no clinically evident ascites. Discrete bimalleolar oedema was present. 

The blood tests revealed a new elevation of the cholestatic enzymes. Compared with the blood tests obtained in the first ED episode, there was a seven-fold increase of total bilirubin, a discrete elevation of ALP, and a four-fold increase of GGT. There was also a slight elevation of cytolytic enzymes (Table 2).

**Table 2:** Blood tests of the second ED episode, revealing cholestatic pattern of the hepatic enzymes, aggravated when compared with the first episode.

	Results	Reference values
Haemoglobin	16.4 g/dL	11.8 - 15.8 g/dL
Leukocytes	8.21x10^9^/L	4-10x10^9^/L
Platelets	141x10^9^/L	150-400x10^9^/L
Urea	109 mg/dL	17-43 mg/dL
Creatinine	1.19 mg/dL	0.7-1.0 mg/dL
Sodium	124 mmol/L	136-145 mmol/L
Potassium	4.9 mmol/L	3.5-5.1 mmol/L
Total bilirubin	17.91 mg/dL	0.3-1.2 mg/dL
Direct bilirubin	12.62 mg/dL	<0.5 mg/dL
Alkaline Phosphatase	519 IU/L	30-120 IU/L
Gamma-glutamyltransferase	1080 IU/L	<38 IU/L
Aspartate Transaminase	108 IU/L	8-35 IU/L
Alanine transaminase	93 IU/L	7-45 IU/L
International Normalized Ratio (INR)	4.62	

**Figure 1:**
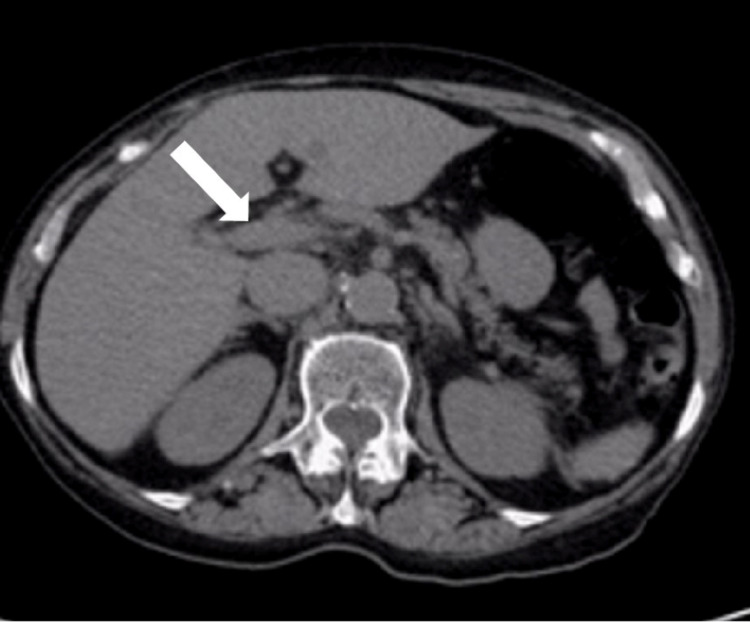
Abdominopelvic CT performed in the emergency department, revealing enlarged hepatic veins (white arrow) and signs of congestive hepatopathy. The exam also excluded the presence of biliary tract obstruction.

An abdominopelvic computed tomography (CT) showed signs of congestive hepatopathy (Figure 1). An abdominal Doppler ultrasound was also performed, showing normal permeability of suprahepatic portal veins and no signs of portal hypertension. Biliary obstruction and ascites were excluded. The patient was admitted for clinical and analytical surveillance and to continue the study. Endoscopic retrograde cholangiopancreatography was performed, excluding signs of biliary obstruction. A magnetic resonance imaging (MRI) of the liver and the biliary ducts was performed, confirming the absence of biliary tract obstruction and showing no signs suggestive of neoplastic disease of the liver. Viral hepatitis due to hepatitis B and C virus infection were excluded. Cytomegalovirus infection was also excluded. An autoimmune study was also performed, revealing negative anti-mitochondrial antibodies, negative antinuclear antibodies (ANA), negative anti-liver-kidney microsomal (LKM) antibodies, and negative smooth-muscle antibodies, which made an auto-immune cause very unlikely.

**Figure 2:**
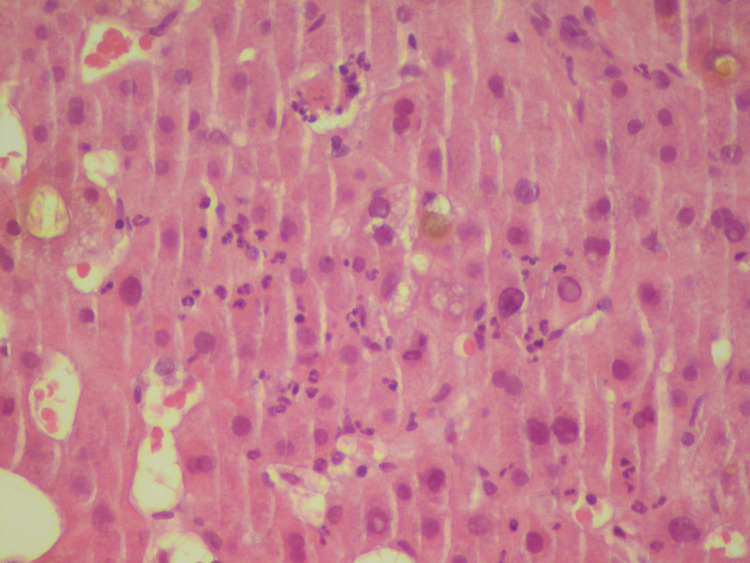
Hepatic biopsy performed one week after starting empirical corticotherapy and acetylcysteine. The biopsy showed a pattern of acute hepatitis, with mainly lobular and periportal inflammation, associated with hepatocyte necrosis and cholestasis of hepatocellular predominance. These alterations were compatible with the hypothesis of drug-induced liver injury (DILI).

After excluding the main causes of alteration of hepatic enzymes (obstructive, neoplastic, infectious, and autoimmune), a diagnosis of probable DILI due to bioflavonoids treatment was assumed. Due to the severe alteration of the cholestatic enzymes, empirical treatment with prednisolone (1 mg/kg of body weight) and acetylcysteine was started. One week after starting the empirical treatment, a hepatic biopsy was performed, revealing a pattern compatible with the clinical hypothesis of DILI (Figure 2).

**Figure 3:**
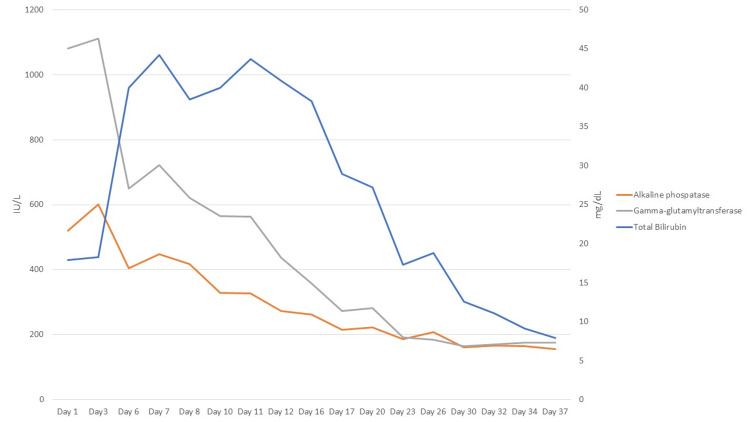
Analytical evolution of cholestatic enzymes (total bilirubin, ALP, and GGT) since observation in the ED department. ALP: alkaline phosphatase; GGT: gamma-glutamyltransferase.

After corticotherapy and acetylcysteine were started, a clinical and analytical improvement was evident. Jaundice started to regress, and pruritus started to subdue. The analytical evolution of total bilirubin, ALP, and GGT is presented in Figure 3. After three weeks of treatment, tapering of prednisolone was started until stoppage, and acetylcysteine was suspended, without recurrence of symptoms or new elevation of hepatic enzymes. 

## Discussion

DILI is a very challenging diagnosis as it can mimic acute and chronic liver diseases, with many different aetiologies; symptoms are nonspecific, varying from fever and nausea to jaundice and right quadrant pain [2].

The first step when suspecting DILI is to characterize its phenotype. Acute DILI is usually identified using biochemical criteria, which include one of the following: an alanine aminotransferase (ALT) elevation of at least five times the upper limit of normal (ULN), an ALP elevation at least two times ULN, or an elevation of ALT of at least three times the ULN plus total bilirubin over tow times the ULN [3]. In patients with abnormal liver tests prior to the event, ULN is replaced with mean baseline values obtained prior to DILI onset [4]. The pattern of hepatic injury is defined by the ratio (R) of ALT (expressed in ULN) divided by ALP (expressed in ULN). Three patterns can be identified using this method: a hepatocellular pattern when R≥5, a cholestatic pattern when R≤2, and a mixed pattern when the R value is between 2 and 5 [4]. In this case, a cholestatic pattern was identified, which is usually more common in older patients [4].

To assist clinicians in the diagnosis of DILI, the Roussel Uclaf Causality Assessment Method (RUCAM) was created [6]. The RUCAM scale provides a more objective evaluation when assessing the possibility of DILI. This scale classifies seven different domains: temporal relationship between exposure to a particular drug and liver injury, analytical evolution of hepatic enzymes after discontinuation of the suspected agent, exclusion of alternative non-drug-related aetiologies, exposure to other medications that could explain DILI, risk factors for the adverse hepatic reaction, evidence in the literature regarding DILI from the drug in question and response to re‐exposure to the medication [3]. The total score ranges from -9 to 10 and classifies the case as highly probable (≥9), probable (6-8), possible (3-5), unlikely (1-2), or excluded (≤0) [6]. In our clinical case, the calculated RUCAM score was 5 (temporal relationship, age as a risk factor and decrease to less than 50% of ALP in less than 180 days), which turns our case into a possible case of DILI. However, the patient had a previous challenge to bioflavonoids that was not evaluated by a physician, clinically evident by the development of jaundice and resolution after the stoppage of the drug. If we assume this situation as a first exposure and the described case as a re-exposure to the drug, the RUCAM score could rise to at least 6, turning our case into a probable DILI. This is a known limitation in the RUCAM score, as it can be difficult to apply in cases where there is missing information [3].

Statin therapy, and more specifically, atorvastatin has been linked with hepatotoxicity, with a mixed pattern of liver injury occurring months after the initiation of the medication [7]. Usually, the liver injury improves after the stoppage of atorvastatin in a few weeks. In this case, atorvastatin was not stopped during evaluation, and an improvement was observed despite the presence of this drug, which excludes this drug as a potential cause for the hepatic injury observed. 

A multitude of drugs have been described as causative of DILI, with a majority being antimicrobial agents, and commonly being used for decades [2,8]. However, to our knowledge, this is the first case of DILI caused by bioflavonoids described in the literature. None other therapeutic agent was initiated in the period described, which makes this drug the most likely to be responsible for the described alterations.

The most important step when treating a suspected DILI is the discontinuation of the suspected agent and avoiding re-exposure to said drug [1,2,8,9]. Some pharmacological therapies seem to have a beneficial effect when started early in the disease progression [4]. N-acetylcysteine is commonly used in the treatment of paracetamol intoxication, but it can have some beneficial effects in other types of DILI. When used early in the course of acute liver failure caused by DILI, it seems to prevent severe encephalopathy and renal failure [4] and better transplant-free survival [1,9]. Corticosteroid therapy is often used as a last resource when all other therapeutic strategies have failed [4,9]. In our clinical case, given the severity of the elevation of the hepatic enzymes, both therapies were empirically started, given that, due to the advanced age and multiple comorbidities, our patient was not an eligible candidate for liver transplantation, as this is the best treatment for severe DILI [1,2,4].

## Conclusions

DILI is a challenging diagnosis because of the multitude of possible differential diagnosis that this clinical entity can mimic. The necessity of a very high suspicion index is needed to stop the causative agent and improve the prognosis of this disease. Our clinical case describes a typical presentation of a DILI with a cholestatic pattern. However, the causative drug (bioflavonoids) has not been yet described as a possible etiologic agent for the development of DILI.
